# Comparative study on the time trends of antimicrobial resistance at animal and human hospitals in a shared community

**DOI:** 10.1016/j.onehlt.2025.101096

**Published:** 2025-05-31

**Authors:** Mackenzie Blackstock, Jieun Park, Matthew Shane Loop, Melanie Hyte, Andrea Perkins, Sarah Grace Gunter, Laura Matthews, Laura Huber

**Affiliations:** a Edward Via College of Osteopathic Medicine - Auburn Campus, 910 S Donahue Dr, Auburn, AL 36832, USA; b Division of Research, Harrison College of Pharmacy, Auburn University, AL 36849, USA; c Department of Health Outcomes Research and Policy, Harrison College of Pharmacy, Auburn University, 362 Thach Concourse, Auburn, AL 36849, USA; d Division of Pharmacotherapy and Experimental Therapeutics, UNC Eshelman School of Pharmacy, University of North Carolina, 301 Pharmacy Ln, Chapel Hill, NC 27516, USA; e Department of Clinical Sciences, College of Veterinary Medicine, Auburn University, 1500 Wire Road, Auburn, AL 36832, USA; f Department of Pharmacy Services, East Alabama Health, 2000 Pepperell Pkwy, Opelika, AL 36801, USA; g Pathobiology Department, College of Veterinary Medicine, Auburn University, 1130 Wire Road, Auburn, AL 36832, USA

**Keywords:** Antimicrobial resistance, Cross-species transmission, Shared community

## Abstract

Antimicrobial resistance (AMR) is a significant global health threat, with increasing morbidity, mortality, and economic consequences. Human and animal health are linked in the spread and evolution of AMR, particularly due to shared antimicrobial usage in healthcare and agriculture. This study investigates the temporal trends in AMR in both a human and an animal hospital within the same community, focusing on five common drug-bug combinations that overlap between these settings. We aim to explore whether AMR patterns in these two hospitals follow similar trajectories, potentially supporting the hypothesis of cross-species transmission of AMR. Data were retrospectively collected from 2010 to 2022 from East Alabama Medical Center (EAMC) and from 2010 to 2023 from Auburn University Veterinary Teaching Hospital (AUVTH). The analysis focused on *Escherichia coli* and *Klebsiella pneumoniae* resistance to cefazolin, ceftriaxone, levofloxacin, and gentamicin, as these were overlapping pairs that had shown statistically significant time trends in preanalysis screening. Bayesian logistic regression was applied to model trends in AMR over time. Generally, the AMR trends in the two hospitals did not align, with decreasing susceptibility in the human hospital and increasing susceptibility in the animal hospital. The trends over time for *K. pneumoniae* susceptibility to gentamicin were the exception, with both the human and animal hospital showing little change over time. Sensitivity analysis that excluded repeated animal isolates revealed stronger alignment between settings in resistance trends, particularly a decreasing sensitivity over time for *E. coli* and ceftriaxone. This finding suggests possible shared environmental factors or interspecies transmission in certain instances. This study emphasizes the need to collect combined data from humans and animals to better understand AMR and create joint efforts for antimicrobial stewardship. Further research is needed to clarify the relationship between AMR in these interconnected environments and inform strategies to mitigate the spread of resistance.

## Highlights

• AMR trends in general do not align between animal and human settings.
• Exceptions exists, especially in *E. coli* susceptibility to ceftriaxone.
• Possible shared environmental factors or interspecies transmission.
• There is a need for combined human and animal data to understand AMR.

## 1 Introduction

The World Health Organization has identified antimicrobial resistance (AMR) as one of the greatest threats to human health [1] and one of the top health challenges facing the 21st century [2,3]. Every year, AMR infections kill 700,000 people, and it is estimated that by 2050, 10 million lives and $300 billion to over $1 trillion of economic output are at risk worldwide [4]. Nationally, 2.8 million human infections and 35,000 deaths are attributable to AMR pathogens annually [5]. Injudicious use of antimicrobials is one cause of AMR, however, pathogen resistance can be acquired from many sources, including food and contact with the environment and animals [[6], [7], [8]]. In 2020, 45 % of U.S. households included dogs and 26 % included cats [9,10]. Companion animals (pets) are often kept in close contact with humans within or near the household for companionship, service, safety, and lifestyle purposes. Medical benefits of having pets include better cardiovascular health, reduced depression, enhanced social development, and helping to establish an exercise routine [11,12]. Humans and pets are both susceptible to infections in various body locations such as the urinary tract, skin, respiratory tract, and oral cavity. Pets are often considered family members and receive a considerable level of healthcare, including frequent antimicrobial use, which contributes to high levels of AMR [13,14].

Several studies have shown bacterial pathogens recovered from pets were found to carry a large range of AMR genes resulting in resistance to critically important antimicrobials [[15], [16], [17], [18], [19], [20]]. Risk of AMR transmission from pets to humans and vice versa is potentially high due to the close proximity between pets and humans within households. Increasing AMR in bacterial pathogens in pets that are opportunistic to humans is an emerging human health concern [[21], [22], [23]], especially for the multidrug resistant-ESKAPE pathogens [24,25], *Enterococcus faecium*, *Staphylococcus aureus*, *Klebsiella pneumoniae*, *Acinetobacter baumannii*, *Pseudomonas aeruginosa* and *Enterobacter* spp. Despite its importance, little is known about the temporality and transmission risks of AMR between animal and human patients within a shared community. The concept of a shared community is critical in understanding AMR dynamics because it highlights the interconnectedness between humans and animals that often occurs in settings where individuals and their pets or livestock live in close proximity or interact regularly. These shared environments can serve as reservoirs for resistant organisms that circulate between species. Such knowledge is essential for designing more effective, integrated antimicrobial stewardship policies and interventions that address AMR in both human and animal populations. In this study, we aim to explore the temporal trends of AMR in both human and animal hospitals within a shared community. This was an exploratory analysis intended to identify whether temporal AMR patterns align across species, which could suggest a potential for interspecies transmission. We did not have access to patient-level data linking individual human and animal cases (e.g., pet ownership), nor were clinical histories available to examine associations between AMR and specific disease presentations or treatments. We hypothesize that resistant organisms may be exchanged between animals and humans through shared environments, such as households, and subsequently introduced into clinical settings. This conceptual framework is reflected in our focus on community-level AMR dynamics rather than spatial proximity. By identifying potential parallels in AMR trends between human and animal clinical settings, we aim to inform the design of future studies that can more directly investigate transmission pathways, shared risk factors, and clinical correlations. This study stands as one of the few in the literature to explore the time trend of AMR in both animals and humans, particularly within a shared community. By shedding light on these crucial interconnections, it paves the way for future collaborations and the inclusion of additional clinics, further advancing research on the transmission dynamics of AMR in a One Health context.

## 2 Methods

### 2.1 Study design and data collection

This was a retrospective comparative study examining AMR trends over time in a human hospital and an animal hospital located within the same community, and approximately 9 miles apart. The data for this study were collected from both hospitals from 2010 to 2023, during which time antimicrobial susceptibility testing was routinely performed on bacterial isolates. The human hospital provided aggregate data for isolates from patients treated in the hospital, while the animal hospital provided patient-level data, including information on species (small vs. large animals) and sample source (e.g., blood, urine, feces). We focused on five common drug-bug combinations that had shown statistically significant time trends in a prior analysis, overlapped between both hospitals, and represented the most commonly tested organisms.

**Supplemental Fig. 1:**
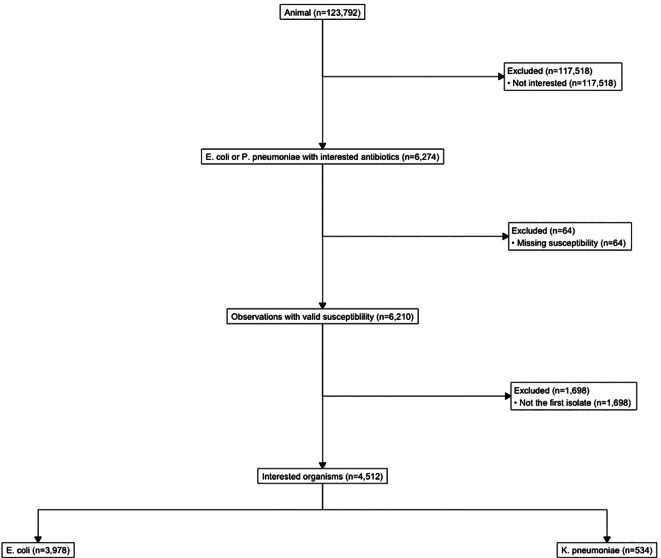
CONSORT diagram for initial isolate inclusion from the animal hospital.

Resistance data was retrospectively collected from antibiogram data from the animal hospital, Auburn University Veterinary Teaching Hospital (AUVTH), and from the human hospital, East Alabama Medical Center (EAMC). Antimicrobial susceptibility testing was performed using automated systems at both hospitals: EAMC utilized the MicroScan WalkAway system (Beckman Coulter, Brea, CA), while AUVTH employed the VITEK® 2 system (bioMérieux, Marcy-l'Étoile, France). Both platforms determine Minimum Inhibitory Concentrations (MICs) using the broth microdilution method, and were previously found to yield comparable results [26]. Data was collected by manual search of cumulative hospital antibiograms for isolate members of the ESKAPE multidrug resistant pathogens which include *E. faecium*, *Proteus mirabilis*, *P. aeruginosa*, *K. pneumoniae*, *S. aureus*, plus *E. coli, Enterococcus faecalis*, and *Staphylococcus intermedius*. Methods for isolation and susceptibility testing for isolates included in cumulative hospital antibiograms followed CLSI guidelines. Antimicrobial susceptitibility in the human and animal hospital included amikacin, amoxicillin-clavulanic acid, ampicillin, cephalosporins (cefovecin, ceftriaxone, cefpodoxime, ceftazidime), clindamycin, doxycycline, enrofloxacin (animal hospital only), ciprofloxacin, levofloxacin, erythromycin, gentamicin, marbofloxacin (animal hospital only), oxacillin, penicillin, nitrofurantoin, trimethoprim/sulfamethoxazole, and vancomycin. The data from the human hospital was recorded as proportion of isolates tested per year, aggregated across patients, and antibiogram data was reflective of cultures from all clinical sources (e.g., blood, urine, sputum). Data from all inpatient units and outpatient locations were included, with deduplication criteria that only allowed for the first isolate per patient per year or per 30 days. Data from the animal hospital were more granular, with isolates recorded at the patient level. Each animal's species (small or large) and the source of the sample (e.g., urine, wound, respiratory) were noted. A sensitivity analysis was conducted using only the first isolate per patient. The CONSORT diagram (Supplemental Fig. 1) demonstrates the workflow for the primary analyses with the inclusion criterea for isolates. Moreover, since animals often receive multiple rounds of testing over time, some animals had repeated culturing and susceptibility. To avoid the impact of repeated measures from the same animals, duplicate identification numbers were excluded from the statistical analyses.

The five drug-bug combinations used in both the human and animal hospitals were:

1. *E. coli* and cefazolin;
2. *E. coli* and ceftriaxone;
3. *E. coli* and levofloxacin;
4. *K. pneumoniae* and gentamicin; and
5. *K. pneumoniae* and levofloxacin

These specific combinations were chosen due to their clinical relevance, as they involve organisms frequently associated with antimicrobial resistance (AMR) [27]. Additionally, the selected compounds are commonly used at both institutions, enabling a direct comparison of resistance trends between the two hospital populations.

### 2.2 Statistical analysis

In both human and animal hospitals, the proportion of isolates that were susceptible to each antimicrobial agent was calculated for each year. Descriptive plots with the proportion of susceptible isolates per year with 95 % confidence bands for the animal and human hospital were created. For the animal hospital, additional descriptive plots were created comparing large versus small species and type of sample.

#### 2.2.1 Bayesian logistic regression for human hospital data

The general modeling strategy was to fit separate models for the human and animal hospital data, and then compare the estimated trend lines between the two models. This separate modeling strategy was used because of the different data structures of the human (aggregated across patients) versus animal (patient-level) datasets. In a sensitivity analysis, we explored the effect of accounting for repeated isolates on the same animal hospital patient to match the deduplication criteria from human samples which only allowed for the first isolate per patient per year or per 30 days to be included.

For the human hospital data, Bayesian logistic regression was used to model the proportion of susceptible isolates over time. The model included year as a continuous predictor variable to capture trends in susceptibility over the study period. The intercept was given a prior distribution of Gaussian (mean = 0, standard deviation = 2.5) and the prior for the year coefficient was Gaussian (mean = 0, standard deviation = 0.64), with the provided prior for the coefficient being automatically scaled by the model software to reflect the nature ranges of the variables. The model was fit using the rstanarm package [28] in R [29], which implements Bayesian inference with Hamiltonian Monte Carlo sampling to estimate the posterior distributions of model parameters. We then plotted the estimated percentage of susceptible isolates for each drug-bug combination, along with 95 % credible intervals (CIs), to visually assess changes in susceptibility over time.

#### 2.2.2 Bayesian logistic regression for animal hospital data

Given that the animal hospital data were collected at the patient level and included repeated measures from the same animals, several modeling strategies were employed. In the primary analysis, all isolates were treated as independent observations, assuming no correlation between repeated isolates from the same animal. A Bayesian logistic regression model with year as a continuous predictor was used, with the same priors as for the human data for almost all the drug-bug combinations except for *E. coli*-cefazolin and *E. coli*-ceftriaxone, which had slightly larger standard deviations. This approach allowed for a straightforward comparison of trends between the two hospitals but did not account for the correlation of susceptibility among repeated isolates on the same patient, which may create overly confident credible intervals. We conducted a sensitivity analysis to account for the correlation among these repeated measures using generalized estimating equations (GEE) with an exchangeable covariance structure. We also conducted a second sensitivity analysis in which we restricted the animal hospital data to only the first isolate collected for each animal, eliminating the impact of repeated measures, and re-fit the same Bayesian logistic regression models as the primary analysis. This approach ensured that the analysis was based on independent observations and allowed for a simpler comparison to the human hospital data. Repeated sampling of the same patient in the animal hospital may have been more often performed on persistent infections that were resistant to prior antibiotics, so the likelihood of susceptibility for the first isolates in the animal patients may have been systematically different than subsequent isolates on the same patient.

#### 2.2.3 Comparison of trends between human and animal hospitals

To compare the temporal trends in AMR between the human and animal hospitals, we plotted the estimated regression lines from each model. We then compared the estimated slopes between the human and animal hospital for each of the regression lines for drug-bug combinations. We estimated the probability that the slopes were in the same direction in both hospitals using the posterior distributions of the year coefficients from each of the respective Bayesian models. We calculated these probabilities for both the primary analysis and the second sensitivity analysis with only the first isolate per patient in the animal hospital included to assess the robustness of our findings. Because the sensitivity analysis using GEE was not fit using Bayesian methods, this comparison was not possible.

## 3 Results

### 3.1 Descriptive statistics

A total of 60,423 *E. coli* isolates from the human hospital, and 5522 from the veterinary hospital between years 2010 to 2023 were analyzed and included in this report. Testing for cefazolin and ceftriaxone in animal hospitals started later, therefore our data included only years 2015 to 2023 for these drug-bug combinations. For *K. pneumoniae*, a total of 10,454 isolates were included in the human hospital and 688 in the animal hospital. *E. coli* susceptibility to cefazolin, ceftriaxone, and levofloxacin were 86.6 %, 93.9 %, and 77.9 %, respectively in the human hospital and 40.7 %, 76.1, and 78.9 % in the animal hospital. *K. pneumoniae* susceptibility to both gentamicin and levofloxacin was 95 % inf the human hospital and 81.4 and 75.1 % respectively in the veterinary hospital (Table 1). The exact number of isolates included per year from the animal and human hospitals are specified in Suppelemental Table 1.

**Table 1:** Summary of the total number of isolates tested in the human hospital and the number of isolates that were susceptible to the tested antimicrobials.

Organism	Antibiotic	Total Isolates	Susceptible Isolates	Percentage susceptible
Human Hospital				
*E. coli*	Cefazolin	20,141	17,437	86.57 %
*E. coli*	Ceftriaxone	20,141	18,908	93.88 %
*E. coli*	Levofloxacin	20,141	15,679	77.85 %
*K. pneumoniae*	Gentamicin	5227	4983	95.33 %
*K. pneumoniae*	Levofloxacin	5227	4969	95.06 %
Animal Hospital				
*E. coli*	Cefazolin	1723	701	40.68 %
*E. coli*	Ceftriaxone	1518	1155	76.09 %
*E. coli*	Levofloxacin	2281	1799	78.87 %
*K. pneumoniae*	Gentamicin	355	289	81.41 %
*K. pneumoniae*	Levofloxacin	333	250	75.08 %

### 3.2 Antimicrobial susceptibility trends in human and animal hospitals

**Fig. 1:**
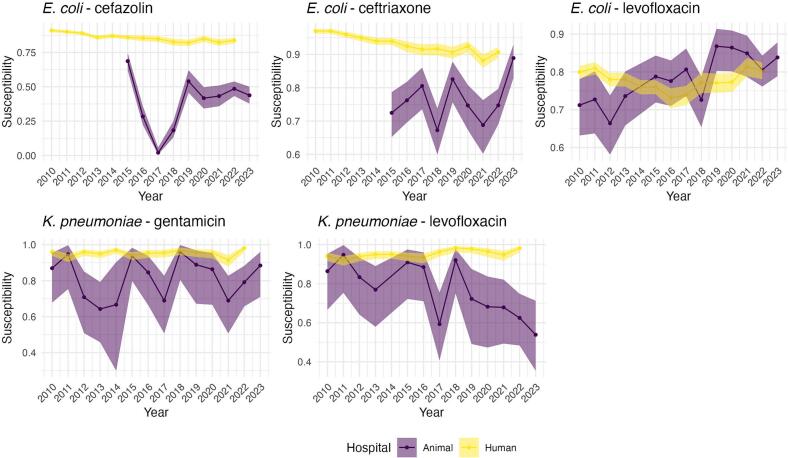
Trend plots of estimated proportions of susceptible isolates and 95 % confidence intervals for each drug-bug combination in each hospital (animal versus human).

**Fig. 2:**
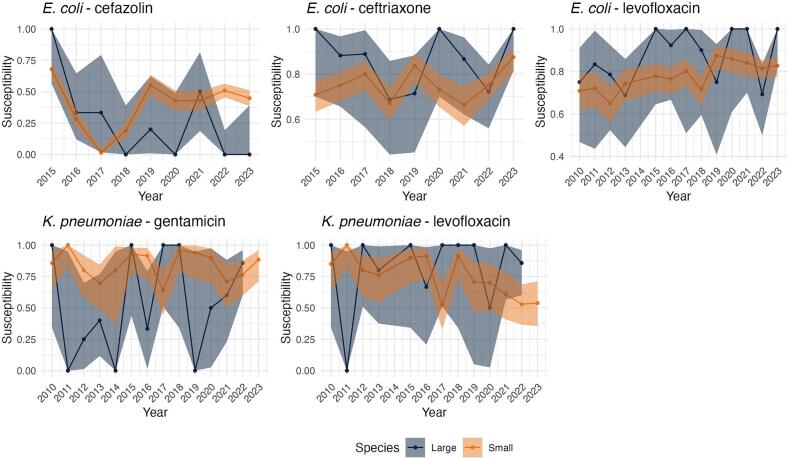
Trend plots of estimated proportions of susceptible isolates and 95 % confidence intervals for each drug-bug combination by animal type (large versus small animals).

The plots of observed susceptibility trends in the human hospital showed that the proportion of *E. coli* isolates susceptible to cefazolin and ceftriaxone decreased over time, while susceptibility to levofloxacin showed a less consistent trend, fluctuating over the study period (Fig. 1). In the isolates from animal origin, there is an increasing trend in susceptibility of *E. coli* to levofloxacin over time and a decreasing trend of *K. pneumoniae* susceptibility to levofloxacin (Fig. 1). All other drug-bug combinations seemed to have an overall constant susceptibility trend with considerable variability between years. Although similar trends are observed, large animal estimated proportion of susceptibility exhibit greater variability than small animal, likely due to the lower number of isolates available for large animals (Figure 2).

### 3.3 Logistic regression results

**Fig. 3:**
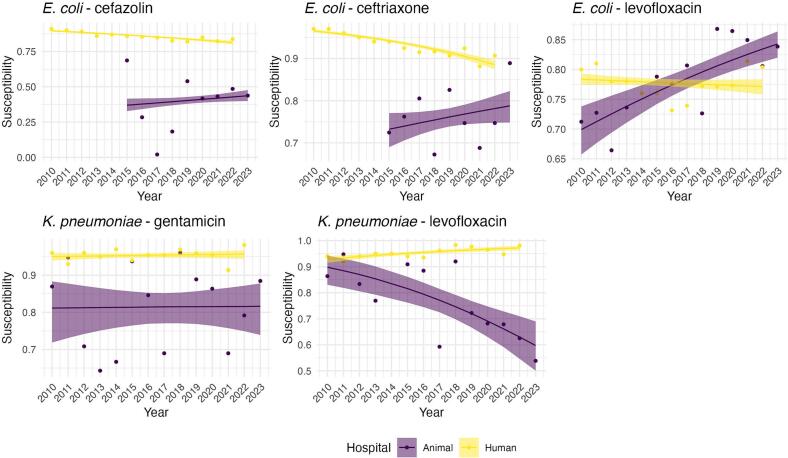
Fitted trend plots for proportion of susceptible isolates over time with 95 % credible intervals. Logistic regression models for animal and human hospitals were fit separately. Repeated observations on the same patient from the animal hospital were treated as independent observations.

In the primary analysis, AMR proportions in the animal hospital were generally more variable than in the human hospital, and the probability that AMR trends moved in the same direction was low for most drug-bug combinations. The exception was *K. pneumoniae* and gentamicin, where the probability of aligned trends was moderately higher (0.528), indicating high uncertainty about whether the trends were in the same direction (Table 2). Based on Fig. 3, this high uncertainty resulted from both trends being flat for the study period. For *E. coli* and levofloxacin, however, the probability was extremely low, indicating that trends in susceptibility between the two hospitals were moving in opposite directions.

**Table 2:** Probabilities that the slopes of the human hospital and animal hospital regression lines are the same.

Organism	Antibiotic	Primary Analysis	First Isolate Only
*E. coli*	Cefazolin	0.029	0.304
*E. coli*	Ceftriaxone	0.044	0.781
*E. coli*	Levofloxacin	0	0.002
*K. pneumoniae*	Gentamicin	0.528	0.567
*K. pneumoniae*	Levofloxacin	0	0.006

**Fig. 4:**
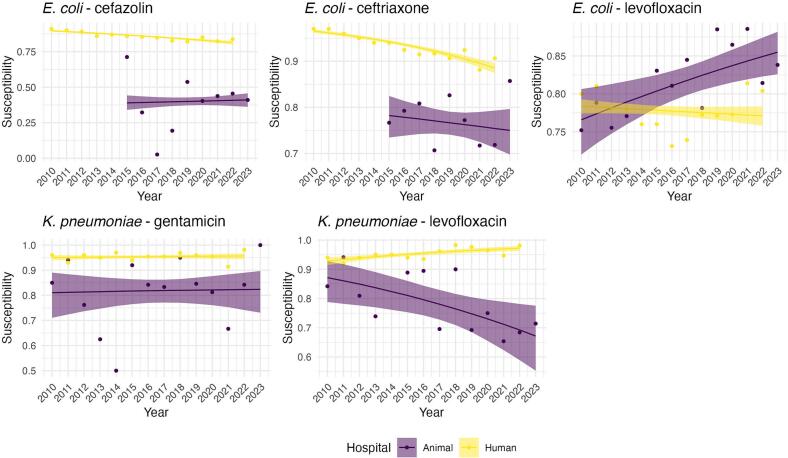
Fitted trend plots for proportion of susceptible isolates over time with 95 % credible intervals. Logistic regression models for animal and human hospitals were fit separately. Only the first observation for each patient in the animal hospital was used.

### 3.4 Sensitivity analyses

**Supplemental Fig. 2:**
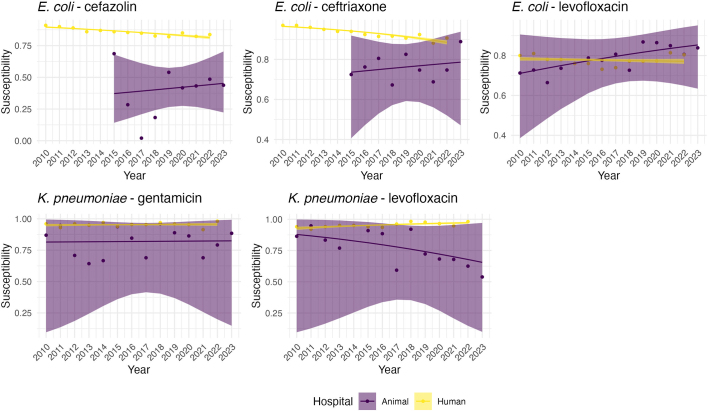
Fitted trend plots for proportion of susceptible isolates over time with 95 % credible intervals. Logistic regression models for animal (purple) and human (yellow) hospitals were fit separately. Repeated observations on the same patient from the animal hospital were accounted for using generalized estimating equations with an exchangeable covariance structure.

When GEE was used to account for the repeated observations among patients in the animal hospital, the trend lines were similar with a much higher uncertainty (Supplemental Fig. 2). When the analysis was restricted to only the first isolate from each animal for the entire period (Fig. 4), the probability that AMR trends were aligned between the human and animal hospitals increased for the *E. coli* – cefazolin combination and the *E. coli* – ceftriaxone combination. This approach helped mitigate potential biases introduced by repeated sampling from the same animals, which likely had initial lack of susceptibility to a given drug-bug combination, which could skew the results. When examining *E. coli* susceptibility to ceftriaxone, the probability that the trends in susceptibility were in the same direction between the two hospitals was 78 % (Table 2). This higher probability suggests that when repeated isolates were excluded, there was a stronger correlation in some AMR trends, particularly for *E. coli*.

## 4 Discussion

This study explored whether temporal trends in AMR in a human hospital mirrored those in an animal hospital within the same community. The central hypothesis was that shared environmental and microbial exposures could lead to similar AMR patterns across species, potentially supporting the idea of cross-species transmission of resistant organisms. The study analyzed data from two hospitals—one human and one veterinary—spanning from 2010 to 2023 and provided new insights into the complex dynamics of AMR development in both human and animal populations. For most drug-bug combinations, the probability that AMR trends moved in the same direction was low, suggesting that AMR development in these two settings does not follow parallel trajectories. For example, *E. coli* and *K. pneumoniae* exhibited differing resistance patterns to cefazolin, ceftriaxone, and levofloxacin between the hospitals. These differences likely reflect variations in antimicrobial usage, infection control practices, and the epidemiology of resistant strains in each setting. This finding is consistent with previously reported [30], that AMR dynamics within human and animal populations tend to be shaped more by sector-specific practices than by direct interspecies transmission. Their large-scale analysis of *E. coli* isolates from humans and animals found that although resistance to some critically important antimicrobials remained low, the resistance trajectories often diverged between species, influenced by differing antimicrobial use patterns and regulatory frameworks. Despite this overall lack of common trend between species in our study, we did identify some areas of alignment. Specifically, when excluding repeated observations on the same patient in the animal hospital, there was a stronger alignment in AMR trends for *E. coli* and ceftriaxone between the two hospitals (78 % probability of aligned trends). This result supports the hypothesis that resistance patterns for certain antibiotics may evolve similarly in both human and animal settings, potentially due to shared environmental factors like common antibiotic use in animal and human health. This aligns with findings from another previous study [31], which demonstrated a relationship between antimicrobial consumption and resistance across humans and animals. Although that study was ecological and did not track clinical isolates longitudinally, it emphasized that increased antibiotic use in either sector was associated with higher AMR prevalence in the other, suggesting environmental or indirect transmission mechanisms may underlie these patterns. Companion animals, such as pets, are an often overlooked source of AMR in humans due to close human-animal contact [32]. A key example is vancomycin-resistant *Enterococcus faecium* (VRE), initially linked to agriculture but later traced to dogs in Denmark [[33], [34], [35]]. Despite bans on certain antibiotics in agriculture, VRE persisted in hospitals, indicating potential pet-to-human transmission. Other cases include *Campylobacter* infections from puppies and methicillin-resistant *Staphylococcus aureus* (MRSA) transmission from pets to humans [36,37]. These cases highlight the importance of developing combined surveillance in human and animal hospitals to track AMR trends. Such surveillance could help identify potential transmission events early and curtail the spread of AMR, providing a proactive approach to managing AMR under the One Health concept. The significance of shared resistomes between species has also been highlighted in metagenomic studies [38] which identified shared antimicrobial resistance genes (ARGs) between human and food animal gut microbiomes. While our study did not include genomic analysis, it underscores the need for future investigations that integrate genomic information and resistome profiling to better assess the extent of ARG sharing in local contexts.

Several limitations must be considered when interpreting these results. First, the drug-bug combinations are narrow and do not encompass the full breadth of possible drug-bug combinations that hold clinical relevance to both people and animals in a shared community. While an inherent limitation of a retrospective study includes lack of control over quality and quantity of historical data sets, this study was still able to identify at least five clinically relevant drug-bug combinations that overlapped across the human and animal hospital with sufficient data to demonstrate statistically significant time trends. Another key issue was the difference in data collection methods between the hospitals. In the human hospital, data were aggregated, which may have obscured variability in resistance patterns at the individual level and masked within-patient variability. Aggregation also limits the ability to adjust for individual patient factors. In contrast, data from the animal hospital were collected at the patient level, offering a more detailed analysis but introducing potential biases from repeated testing of animals with chronic infections. Additionally, the animal hospital had fewer isolates (5522) compared to the human hospital (60,423), limiting our ability to draw firm conclusions. Finally, testing of certain antibiotics began later in the animal hospital, reducing available data to analyze AMR trends over time. Moreover, we did not have access to detailed clinical or epidemiological data—such as pet ownership, cohabitation status, or treatment histories—that would allow direct linkage between human and animal patients. The lack of this information limits the ability to confirm transmission pathways or assess shared risk factors. Future studies would benefit from integrated data collection across sectors to provide a clearer picture of the interplay between human and animal AMR trends.

Despite limitations, this study offers valuable insights into AMR dynamics in human and animal healthcare settings. The lack of alignment in AMR trends points to complex factors like antimicrobial practices, infection control, species-specific influences, and environmental factors. At the same time, isolated areas of convergence—especially regarding *E. coli* and ceftriaxone—suggest that shared community-level exposures may influence AMR in both sectors under certain conditions. Further research is needed to explore shared drivers of AMR and assess the role of cross-species transmission in AMR development. Longitudinal studies with larger samples and refined data collection are crucial for clarifying the relationship between AMR trends in these settings. Integrated antimicrobial stewardship, coordinated surveillance, and targeted interventions across human and veterinary systems are essential to combat AMR and zoonotic transmission.

## 5 Conclusion

This study demonstrates that AMR trends in human and animal hospitals within the same community were mostly different for the drug-bug combinations we studied. While the findings suggest that AMR development in these settings may be driven by different factors, there is some evidence of temporal overlap in resistance patterns for certain drug-bug combinations. This hypothesis-generating study highlights the potential for cross-species transmission of multidrug-resistant (MDR) organisms within shared environments and underscores the need for future genomic investigations. Whole-genome sequencing will be essential in elucidating the genetic relatedness of isolates and identifying specific resistance determinants, particularly for cephalosporins, where different resistance genes may influence interpretation. These findings support the importance of further research into the complex ecological and microbiological drivers of AMR in both human and animal populations to inform targeted mitigation strategies. By improving our understanding of the factors driving AMR, we can develop more effective strategies to combat this growing public health threat.

The following is the supplementary data related to this article.

Supplemental Table 1 Annual antibiotic susceptibility of *Escherichia coli* and *Klebsiella pneumoniae* isolates from human and animal sources (2010−2023). Supplemental Table 1

## CRediT authorship contribution statement

**Mackenzie Blackstock:** Writing – review & editing, Writing – original draft, Investigation, Conceptualization. **Jieun Park:** Writing – review & editing, Visualization, Validation, Software, Resources, Methodology, Formal analysis, Data curation. **Matthew Shane Loop:** Writing – review & editing, Visualization, Validation, Software, Resources, Methodology, Formal analysis, Data curation. **Melanie Hyte:** Writing – review & editing, Software, Resources, Methodology, Investigation, Data curation, Conceptualization. **Andrea Perkins:** Writing – review & editing, Resources, Methodology, Investigation, Data curation, Conceptualization. **Sarah Grace Gunter:** Writing – review & editing, Software, Resources, Methodology, Investigation, Data curation, Conceptualization. **Laura Matthews:** Writing – review & editing, Software, Resources, Conceptualization. **Laura Huber:** Writing – review & editing, Writing – original draft, Validation, Supervision, Software, Resources, Project administration, Methodology, Investigation, Data curation, Conceptualization.

## Declaration of generative AI and AI-assisted technologies in the writing process

During the preparation of this work the author (Huber, L.) used ChatGPT in order to improve readability of some parts of the manuscript. After using this tool/service, the author(s) reviewed and edited the content as needed and take(s) full responsibility for the content of the publication.

## Declaration of competing interest

The authors declare that they have no known competing financial interests or personal relationships that could have appeared to influence the work reported in this paper.

## Data Availability

Data not available due to confidentiality.
