# Supplementary material for: Comparative study on the time trends of antimicrobial resistance at animal and human hospitals in a shared community
Source: One Health. 2025 May 31;20:101096. doi: 10.1016/j.onehlt.2025.101096 (PMC12173080; doi:10.1016/j.onehlt.2025.101096)
Supplement: Supplemental Table 1 — Annual antibiotic susceptibility of Escherichia coli and Klebsiella pneumoniae isolates from human and animal sources (2010−2023). [file mmc3.docx]

|  | ***E. coli*** | | | | | | ***K. pneumoniae*** | | | | |
| --- | --- | --- | --- | --- | --- | --- | --- | --- | --- | --- | --- |
|  | **Cefazolin** | | **Ceftriaxone** | | **Levofloxacin** | |  | **Gentamicin** | | **Levofloxacin** | |
| **Year** | Human | Animal | Human | Animal | Human | Animal | **Year** | Human | Animal | Human | Animal |
| **2010** | 90.98% (2,037/2,239) |  | 97.01% (2,172/2,239) |  | 79.99% (1,791/2,239) | 71.22% (99/139) | **2010** | 95.98% (454/473) | 86.96% (20/23) | 94.08% (445/473) | 86.36% (19/22) |
| **2011** | 90.01% (1,974/2,193) |  | 96.99% (2,127/2,193) |  | 80.98% (1,776/2,193) | 72.73% (80/110) | **2011** | 92.96% (462/497) | 94.74% (18/19) | 91.95% (457/497) | 94.74% (18/19) |
| **2012** | 89% (1,926/2,164) |  | 95.98% (2,077/2,164) |  | 78% (1,688/2,164) | 66.42% (91/137) | **2012** | 95.95% (474/494) | 70.83% (17/24) | 93.93% (464/494) | 83.33% (20/24) |
| **2013** | 85.98% (1,717/1,997) |  | 94.99% (1,897/1,997) |  | 78.02% (1,558/1,997) | 73.61% (106/144) | **2013** | 94.94% (450/474) | 64.29% (18/28) | 94.94% (450/474) | 76.92% (20/26) |
| **2014** | 87.01% (1,694/1,947) |  | 93.99% (1,830/1,947) |  | 76.01% (1,480/1,947) |  | **2014** | 97.01% (487/502) | 66.67% (4/6) | 95.02% (477/502) |  |
| **2015** | 86% (1,757/2,043) | 68.66% (138/201) | 93.98% (1,920/2,043) | 72.46% (121/167) | 76.02% (1,553/2,043) | 78.79% (130/165) | **2015** | 94.07% (428/455) | 93.75% (30/32) | 94.07% (428/455) | 90.91% (20/22) |
| **2016** | 85.39% (947/1,109) | 28.4% (48/169) | 92.43% (1,025/1,109) | 76.22% (141/185) | 73.13% (811/1,109) | 77.6% (142/183) | **2016** | 95.34% (348/365) | 84.62% (22/26) | 93.42% (341/365) | 88.46% (23/26) |
| **2017** | 84.84% (968/1,141) | 2.03% (3/148) | 91.5% (1,044/1,141) | 80.52% (124/154) | 73.88% (843/1,141) | 80.67% (121/150) | **2017** | 95.47% (379/397) | 68.97% (20/29) | 96.22% (382/397) | 59.26% (16/27) |
| **2018** | 82.65% (762/922) | 18.29% (30/164) | 91.65% (845/922) | 67.24% (117/174) | 77.22% (712/922) | 72.62% (122/168) | **2018** | 96.92% (283/292) | 96% (24/25) | 98.29% (287/292) | 92% (23/25) |
| **2019** | 82.06% (878/1,070) | 53.96% (75/139) | 90.65% (970/1,070) | 82.55% (123/149) | 77.1% (825/1,070) | 86.81% (125/144) | **2019** | 95.81% (343/358) | 88.89% (16/18) | 97.77% (350/358) | 72.22% (13/18) |
| **2020** | 85.04% (921/1,083) | 41.72% (63/151) | 92.43% (1,001/1,083) | 74.68% (118/158) | 77.29% (837/1,083) | 86.45% (134/155) | **2020** | 95.45% (273/286) | 86.36% (19/22) | 96.5% (276/286) | 68.18% (15/22) |
| **2021** | 82.31% (814/989) | 43.21% (70/162) | 88.17% (872/989) | 68.8% (86/125) | 81.4% (805/989) | 84.94% (141/166) | **2021** | 91.56% (282/308) | 68.97% (20/29) | 94.81% (292/308) | 67.86% (19/28) |
| **2022** | 83.76% (1,042/1,244) | 48.53% (165/340) | 90.68% (1,128/1,244) | 74.7% (189/253) | 80.39% (1,000/1,244) | 80.56% (290/360) | **2022** | 98.16% (320/326) | 79.17% (38/48) | 98.16% (320/326) | 62.5% (30/48) |
| **2023** |  | 43.78% (109/249) |  | 88.89% (136/153) |  | 83.85% (218/260) | **2023** |  | 88.46% (23/26) |  | 53.85% (14/26) |
| **Total** | 86.57% (17,437/20,141) | 40.68% (701/1,723) | 93.88% (18,908/20,141) | 76.09% (1,155/1,518) | 77.85% (15,679/20,141) | 78.87% (1,799/2,281) | **Total** | 95.33% (4,983/5,227) | 81.41% (289/355) | 95.06% (4,969/5,227) | 75.08% (250/333) |

**Supplemental Table 1:** Annual antibiotic susceptibility of Escherichia coli and Klebsiella pneumoniae isolates from human and animal sources (2010–2023).
